# Machine Learning Analyzed Weather Conditions as an Effective Means in the Predicting of Acute Coronary Syndrome Prevalence

**DOI:** 10.3389/fcvm.2022.830823

**Published:** 2022-04-08

**Authors:** Aleksandra Wlodarczyk, Patrycja Molek, Bogdan Bochenek, Agnieszka Wypych, Jadwiga Nessler, Jaroslaw Zalewski

**Affiliations:** ^1^Department of Coronary Artery Disease and Heart Failure, Jagiellonian University Medical College, John Paul II Hospital, Kraków, Poland; ^2^Institute of Meteorology and Water Management, National Research Institute, Warsaw, Poland; ^3^Department of Climatology, Jagiellonian University, Kraków, Poland

**Keywords:** weather, acute coronary syndrome, myocardial infarction, machine learning, artificial intelligence, prediction

## Abstract

**Background:**

The prediction of the number of acute coronary syndromes (ACSs) based on the weather conditions in the individual climate zones is not effective. We sought to investigate whether an artificial intelligence system might be useful in this prediction.

**Methods:**

Between 2008 and 2018, a total of 105,934 patients with ACS were hospitalized in Lesser Poland Province, one covered by two meteorological stations. The predicted daily number of ACS has been estimated with the Random Forest machine learning system based on air temperature (°C), air pressure (hPa), dew point temperature (Td) (°C), relative humidity (RH) (%), wind speed (m/s), and precipitation (mm) and their daily extremes and ranges derived from the day of ACS and from 6 days before ACS.

**Results:**

Of 840 pairwise comparisons between individual weather parameters and the number of ACS, 128 (15.2%) were significant but weak with the correlation coefficients ranged from −0.16 to 0.16. None of weather parameters correlated with the number of ACS in all the seasons and stations. The number of ACS was higher in warm front days vs. days without any front [40 (29–50) vs. 38 (27–48), respectively, *P* < 0.05]. The correlation between the predicted and observed daily number of ACS derived from machine learning was 0.82 with 95% CI of 0.80–0.84 (*P* < 0.001). The greatest importance for machine learning (range 0–1.0) among the parameters reached Td daily range with 1.00, pressure daily range with 0.875, pressure maximum daily range with 0.864, and RH maximum daily range with 0.853, whereas among the clinical parameters reached hypertension daily range with 1.00 and diabetes mellitus daily range with 0.28. For individual seasons and meteorological stations, the correlations between the predicted and observed number of ACS have ranged for spring from 0.73 to 0.77 (95% CI 0.68–0.82), for summer from 0.72 to 0.76 (95% CI 0.66–0.81), for autumn from 0.72 to 0.83 (95% CI 0.67–0.87), and for winter from 0.76 to 0.79 (95% CI 0.71–0.83) (*P* < 0.001 for each).

**Conclusion:**

The weather parameters have proven useful in predicting the prevalence of ACS in a temperate climate zone for all the seasons, if analyzed with an artificial intelligence system. Simultaneously, the analysis of individual weather parameters or frontal scenarios has provided only weak univariate relationships. These findings will require validation in other climatic zones.

## Introduction

When I look out the window, I can forecast whether today's see of practice will be busy or not. This unwritten, intuitive observation often works in the case of an interventional cardiologist treating patients with acute coronary syndromes (ACS). Nevertheless, this common truth is insufficiently proven and does not allow for any practical recommendations to be drawn. A seasonal variation in the prevalence of acute coronary syndromes (ACS) and cardiovascular morbidity is a conventional wisdom. Already in 1926, an association was noted between coronary thrombosis and cold weather in New England during winter (1).

Although weather is a complex phenomenon, usually the impact of only individual weather parameters on ACS prevalence has been analyzed so far. Bayentin et al. (2) have shown that cold temperatures during winter months and hot periods during the summer are associated with a 12% increase in the daily hospital admission rate for ischemic heart disease. In a large Swedish registry, one incorporating more than 280,000 patients, an increase in minimum air temperature by 7.4°C was associated with the reduction of the prevalence of the ACS ratio by 2.8% (3). In contrast, Schwartz et al. have revealed that with each 1°C temperature decrease the mortality ratio rises by 0.49% and the frequency of all-cause, circulatory, coronary heart disease, and ST-segment elevation myocardial infarction (STEMI) death (4). Blazejczyk et al. (5) have shown that the number of deaths attributed to a strong heat stress was five times higher and the total mortality was almost 10% higher in June 2019 than the average for the period of 2010–2018 in Poland. Also, the exposure to an acute air pressure decrease more than 10.7 hPa within 7 days before STEMI increased the rate of STEMI (odds ratio 1.12, 95% CI 1.03–1.21) (6). In the population of New York state, certain demographic groups including the elderly, males, people with Medicaid insurance, people living in warmer areas, or in areas with a high PM2.5 concentration were more prone to cold acute myocardial infarction (MI) effects than others (7). Barnett et al. (8) have proved that inhabitants of colder countries such as North Sweden, North Korea, or Finland are less prone to ACS with temperature changes. This is most likely caused by better protection against cold temperature including clothes as well as housing and metabolic adaptation by inhabitants of these cold areas as observed in the Indians of Tierra del Fuego, Arctic Indians, and Inuits (9). In turn, in Poland, an increase in mortality by 9–19% on cold stress days was noted mainly in the so-called “cool” cities characterized by a clear thermal optimum, approximately in the range of 5–30°C of the Universal Thermal Climate Index (UTCI) (10).

Despite a lot of univariate associations between weather parameter and the prevalence of ACS, their prediction in a given geographical or climatic zone and season is so far ineffective. On the other hand, data concerning a multifactorial approach to this issue are limited (11). Data mining and machine learning techniques seem to be the optimal choices for the reasons that they are trying to predict non-linear and complex relationships between many parameters (12, 13). Among algorithms mostly used in meteorological and climatological studies, the Random Forest method is widely applied (14, 15). Therefore, we sought to investigate if multiple weather conditions analyzed together with an artificial intelligence system are useful in predicting the prevalence of ACS in a temperate climate zone. Lesser Poland Province, being the area of interest, is located in Central Europe where the global circulation model constitutes a westerly advection dependent also on the low pressure systems allocated in north-west Europe in both a cold and warm half-year.

## Methods

### Study Design and Population

Between 1 January 2008 and 31 December 2018, a total of 105,934 patients with were hospitalized in the north part of Lesser Poland Province. The region of interest had a population of 1,812,272 inhabitants in 2014 and in this period was served by two meteorological stations in Tarnow (Station A, 50.03 N, 20.98 E) and Krakow (Station B, 50.08 N, 19.8 E), both located approximately 200 m above sea level (Supplementary Figure 1). In the area represented by stations A and B, 30,143 and 75,791 of patients with ACS were treated, respectively.

The studied population was composed of patients who had been admitted to emergency medical services, emergency departments, or hospital wards and their diagnosis was conducted by the treating physician and coded at discharge according to the International Classification of Diseases (ICD) classification as unstable angina (UA) (I.20.0), ST-segment elevation myocardial infarction (STEMI) including acute transmural MI of the anterior wall (I21.0), of the inferior wall (I21.1), of other sites (I21.2), or non-ST-segment elevation myocardial infarction (NSTEMI) including acute transmural MI of an unspecified site (I21.3), acute subendocardial MI (I21.4), or unspecified acute MI (I21.9). The exclusion criteria for this study were an age of <18 years and doubled records of the same patient who was transferred between two different units due to the same incident. The clinical characteristics of the studied patients, including age, sex, diabetes mellitus (E10–E14), hypertension (I10–I15), duration of hospitalization, history of renal failure (N18–N19) or stroke (I62–I64), and in-hospital mortality, were obtained from the Polish National Health Fund (NHF) registry. This study protocol complied with the Declaration of Helsinki was approved by the Ethical Committee of the Jagiellonian University (approval number 1072.6120.88.2020).

### Meteorological Data and Definitions

Meteorological data were obtained from the Institute of Meteorology and Water Management-National Research Institute (IMWM-NRI) operating as the National Weather Service. The weather registry included air pressure (P) (hPa), air temperature (T) (°C), dew point temperature (Td) (°C), relative humidity (RH (%), wind speed (WS) (m/s), and precipitation (RR) (mm) as measured in accordance with the World Meteorological Organization's standards and regulations (16). All the data were collected at synoptic stations each hour a day with the use of calibrated instruments, i.e., barometers, dry and wet bulb thermometers, anemometers, and rain gauges, respectively. Most of measurements were in parallel conducted with dedicated automated sensors for further validation. All the meteorological variables, with their daily extremes, including maximum (T_max) or minimum (T_min) temperature, maximum (Td_max) or minimum (Td_min) of dew point temperature, maximum (P_max) or minimum (P_min) of air pressure, maximum (RH_max) or minimum (RH_min) of relative humidity, maximum (WS_max) or minimum (WS_min) of wind speed, and maximum (RR_max) or minimum (RR_min) of precipitation, were analyzed for the day of ACS and for 6 days prior to ACS itself. The daily ranges of the aforementioned variables were defined as the difference between the maximum and minimum value within each analyzed day and expressed as the range of temperature (T_range), range of dew point temperature (Td_range), range of air pressure (P_range), range of relative humidity (RH_range), range of wind speed (WS_range), and range of precipitation (RR_range). To indicate the day prior to the index ACS for which the weather conditions were analyzed included was a subscript with values from 1 to 6. For example, RH_max_2_ means a maximum relative humidity 2 days prior to the index ACS. Furthermore, a 3-h air pressure tendency (P_3h__tend) and a 6-h aggregated amount of precipitation (RR_6h_) were added to the overall analysis. Any negative value of P_3h__tend expresses pressure decrease, whereas any positive value of P_3h__tend expresses pressure increase.

Finally, the catalog of synoptic scenarios in the upper Vistula river basin covering the area of Lesser Poland Province (17) was used to distinguish the type and direction of air masses advection as well as the type of pressure pattern together with the type of frontal system. Synoptic maps also served as the source of information of frontal systems distinguished by a surface weather analysis supported by upper air information (18). Strong horizontal temperature, moisture, wind gradient, as well as the vertical shear of a horizontal wind and high vorticity as the attributes of the boundaries between air masses were identified and every front was classified as cold, warm, quasi-stationary, or occluded.

Weather seasons were established on the basis of meteorological rules as follows: spring (1st March to 31st May), summer (1st June to 31th August), autumn (1st September to 30th November), and winter (1st December to 28/29th February).

### Statistics

Statistical analysis was performed with SPSS Statistics software (version 25.0.0.2, IBM Incorporation, USA). Continuous variables are expressed as median (interquartile range) and categorical variables are expressed as number (percentage). Continuous variables were first checked for normal distribution by the Shapiro–Wilk test. Differences between two continuous variables were compared by the Student's *t*-test or the Mann–Whitney *U* test, if distribution was normal or different than normal, respectively. ANOVA followed by the *post-hoc* Bonferroni test was used to compare the differences in the three or more groups with normally distributed data, whereas non-normally distributed data were analyzed by the Kruskal–Wallis test and the differences between the individual groups were identified using a test for multiple comparisons of mean ranks. Categorical variables were analyzed by the chi-squared test or the Fisher's exact test. The association between two variables with a normal or non-normal distribution was assessed by the Pearson or Spearman test, respectively. A two-tailed *P*-value of < 0.05 was considered as statistically significant.

### Predictions With Machine Learning System

Machine learning analysis was performed using the R package with the Random Forest algorithm (19). We examined models with repeated 10-fold cross-validation (10 repeats), which partitions the original sample into 10 disjoint subsets, uses seven of those subsets in the training process, and then makes predictions about the remaining subset. We trained an ensemble classifier by using the results of a set of constituent classifiers by taking a (weighted) vote of their individual predictions. The predictive learning model was based on artificial intelligence with the Random Forest algorithm. The latter is a decision tree-based method constructed by creating a series of decision trees from bootstrapped training samples (20). The decision tree split is a random process, where a new division of data is constructed, rather than using the full set of predictors. The model takes predictions from individual learning algorithm as an input to build an ensemble learning predictive model that classified required meteorological parameters up to 6 days before the day with a specific number of ACS. The variable importance of each weather parameter was a derivative of all its measurements obtained on the day of ACS and 6 days before ACS. For example, the variable importance for T_max means value calculated for all the maximum temperatures collected within 7 days. The training and testing datasets were driven from meteorological parameters, their derivatives, and were combined with the date and day of the week for each number of ACS, as there was found a decreasing trend in the numbers of ACS between 2008 to 2018, as well as significant differences in their distribution between Monday and Saturday or Sunday. The Random Forest was used to build a predictive model, as well as to give information about the learning process itself. In this study, the variable importance of each parameter was examined to quantify how a meteorological condition or clinical characteristic is important in the prediction of the number of ACS per day. The higher the variable importance value (results ranged from 0 to 1), the more important the given parameter was in the training of the model. The prediction performed with the Random Forest algorithm was finally adjusted for age, sex, diabetes mellitus, arterial hypertension, renal failure, and history of stroke.

## Results

### Study Population

The demographic and clinical characteristics of the studied patients are shown in Table 1. In the whole study period, unstable angina was the most frequent diagnosis comprising 52.7% of all the patients with ACS, while STEMI occurred in fewer than one-fifth of patients (Table 1). Patients with STEMI were the youngest (*P* < 0.001), with the highest percentage being males (*P* < .001). Only 1% of patients with UA died during index hospitalization, whereas the highest in-hospital mortality occurred in the STEMI population (*P* < 0.001). Simultaneously, patients with NSTEMI were characterized by the highest percentage of diabetes mellitus, hypertension, renal failure, and history of stroke (all *P* < 0.001) (Table 1).

**Table 1:** Baseline clinical characteristics.

	**All patients ***n*** = 105,902**	**UA** ***n*** **= 55,786**	**STEMI** ***n*** **= 20,042**	**NSTEMI** ***n*** **= 30,074**	***P*** **-value**
Age, years	68 (60–76)	67 (60–75)	66 (57–76)	70 (61–79)	<0.001
Male	65,859 (62.2)	33,828 (60.6)	13,180 (65.9)	18,851 (62.7)	<0.001
**Comorbidities**					
Diabetes mellitus type 1 or 2	37,419 (35.3)	19,452 (34.9)	6,530 (32.6)	11,437 (38.0)	<0.001
Hypertension	75,418 (71.2)	39,474 (70.6)	13,799 (68.9)	22,145 (73.6)	<0.001
Renal failure	4,798 (4.5)	2,074 (3.7)	804 (4.0)	1,920 (6.4)	<0.001
History of stroke	7,211 (6.8)	3,407 (6.1)	1,432 (7.1)	2,372 (7.9)	<0.001
**Outcome**					
Length of hospitalization, days	6 (3–9)	7 (4–9)	5 (4–9)	5 (3–9)	<0.001
In-hospital death	4,649 (4.4)	583 (1.0)	1,967 (9.8)	2,099 (7.0)	<0.001

*Data are shown as median (interquartile range) or numbers (percentage), ACS, acute coronary syndrome; UA, unstable angina; STEMI, ST elevation myocardial infarction; NSTEMI, non-ST elevation myocardial infarction; P-value for differences between UA, NSTEMI and STEMI*.

The total number of acute coronary syndromes has been decreasing since 2013 (Supplementary Table 1). Between 2008 and 2018, the annual number of UA decreased by 67% and STEMI by 47%. Simultaneously, the absolute number of patients with NSTEMI increased during the analyzed period by 39%. In 2018, hypertension was identified more frequently than in 2008 (68.1 vs. 65.8%, *P* = 0.002, respectively), but diabetes mellitus (30.0 vs. 34.5%, *P* < 0.001), renal failure (2.4 vs. 4.7%, *P* < .001), and a history of stroke (4.0 vs. 7.7%, *P* < 0.001) were identified less frequently. The mortality rate was similar in 2018 vs. 2008 (4.7 vs. 4.6%, *P* = 0.377).

Patients with ACS were hospitalized most frequently on Mondays (*P* < 0.0001, Supplementary Table 2) without significant trends in the annual cycle (Supplementary Figure 2). UA was most frequent on Mondays (*P* < 0.0001), while STEMI (*P* < 0.0001) or NSTEMI (*P* < 0.0001) was most frequent on Saturdays. The oldest patients with a median age of 69 years were admitted on weekends. Also, renal failure (5.1 vs. 4.1%, *P* = 0.002) or stroke (7.3 vs. 6.7%, *P* = 0.002) was more frequent, while in-hospital death was higher (6.7 vs. 3.9%, *P* < 0.001) in patients admitted during weekends as compared with working days.

### General Meteorological Characteristics

Both the stations are located relatively close. So, for all the seasons, most of the weather characteristics had a similar distribution. However, due to differences in their geographical location, the median values of most of the meteorological characteristics calculated for the study period of 11 years were different in both the stations (Table 2). The highest absolute differences between both the stations concerned with air temperature, relative humidity, and wind speed. Station A had higher maximum and minimum temperatures than station B in all the seasons, with the median T_max value in summer reaching 24.9°C as compared to 24.3°C in station B. Station A had lower median values of RH_min also in all the seasons, with the biggest difference for winter. Median values of wind speed in station A were equal to 3 m/s, while for station B they reached 5 m/s for autumn and 6 m/s for the rest of the year (Table 2).

**Table 2:** Seasonal weather characteristics at both the meteorological stations.

**Weather parameter**	**Season**	**Station A**	**Station B**	***P*** **-value**
T_max, °C	Spring	15.1 (9.6–20.4)	14.5 (9.0–19.6)	<0.001
	Summer	24.9 (21.4–28.3)	24.3 (20.9–27.5)	<0.001
	Autumn	14.3 (9.3–19.1)	13.4 (8.7–18.6)	<0.001
	Winter	3.2 (−0.5–6.8)	2.4 (−0.9–5.7)	<0.001
T_min, °C	Spring	4.4 (0.7–8.2)	3.8 (0.1–7.7)	<0.001
	Summer	13.2 (10.8–15.8)	13.1 (10.9–15.5)	0.021
	Autumn	5.7 (2.2–9.3)	4.9 (1.1–8.5)	<0.001
	Winter	−1.9 (−6.2–0.9)	−2.9 (−6.8 to −0.3)	<0.001
P_max, hPa	Spring	1,018 (1,013–1,023)	1,019 (1,013–1023)	<0.001
	Summer	1,018 (1,014–1,020)	1,018 (1,015–1,021)	<0.001
	Autumn	1,021 (1,016–1,026)	1,021 (1,017–1,026)	0.075
	Winter	1,022(1,015–1,028)	1,022 (1,015–1,028)	0.027
P_min, hPa	Spring	1,013 (1,007–1,018)	1,013 (1,007–1,018)	<0.001
	Summer	1,013 (1,010–1,016)	1,014 (1,010–1,016)	<0.001
	Autumn	1,016 (1,011–1,021)	1,016 (1,011–1,021)	0.286
	Winter	1,015 (1,008–1,022)	1,015 (1,008–1,022)	0.255
RH_max, %	Spring	95 (91–98)	95 (91–97)	0.002
	Summer	96 (93–98)	95 (92–97)	<0.001
	Autumn	97 (93–98)	96 (95–98)	<0.001
	Winter	94 (89–97)	96 (93-−98)	<0.001
RH_min, %	Spring	44 (34-60)	47 (37–64)	<0.001
	Summer	44 (37–57)	46 (38–58)	<0.001
	Autumn	61 (49–74)	65 (52–79)	<0.001
	Winter	69 (59–79)	75 (65–83)	<0.001
WS_max, m/s	Spring	3 (3–4)	6 (5–8)	<0.001
	Summer	3 (2–3)	6 (4–7)	<0.001
	Autumn	3 (2–4)	5 (4–7)	<0.001
	Winter	3 (2–4)	6 (4–8)	<0.001

*Data are shown as median (interquartile range), T, daily temperature; P, air pressure; RH, relative air humidity; WS, wind speed;max, maximum daily value; min, minimum daily value; P-value for comparisons between station A and B*.

### Acute Coronary Syndrome Prevalence vs. Individual Weather Parameters

Analysis comparing individual weather parameters derived from the day of ACS and the 6 preceding days with the number of ACS in the index day including their seasonal distribution concerned 420 pairwise comparisons for each station separately. Of them, in station A 88 (21.0%) and in station B 40 (9.5%) were significant but weak, with the correlation coefficients ranged from −0.16 to 0.16 (Table 3 and Supplementary Table 3).

**Table 3:** The correlations between the number of acute coronary syndrome (ACS) and the weather conditions as measured in the day of ACS.

		**Station A**				**Station B**			
		**Spring**	**Summer**	**Autumn**	**Winter**	**Spring**	**Summer**	**Autumn**	**Winter**
T_max °C	r	0.01	−0.06	0.00	**−0.12**	−0.06	−0.03	−0.01	−0.02
	P	0.87	0.06	0.96	**<0.001**	0.07	0.28	0.70	0.61
T_min, °C	r	−0.01	−0.00	−0.06	**−0.14**	−0.06	−0.04	0.02	**−0.07**
	P	0.89	0.93	0.08	**<0.001**	0.07	0.20	0.46	**0.03**
T_range, °C	r	0.01	**−0.07**	0.06	0.04	−0.02	−0.01	−0.04	**0.08**
	P	0.68	**0.03**	0.07	0.17	0.45	0.78	0.18	**0.01**
P_max, hPa	r	0.02	−0.02	0.03	0.06	−0.03	−0.01	−0.04	0.05
	P	0.46	0.56	0.30	0.06	0.30	0.83	0.17	0.13
P_min, hPa	r	0.02	−0.03	0.02	0.02	−0.03	−0.02	−0.03	0.04
	P	0.55	0.29	0.45	0.57	0.37	0.50	0.31	0.27
P_range, hPa	r	0.01	0.04	0.01	**0.08**	−0.01	0.03	−0.02	0.02
	P	0.79	0.25	0.70	**0.02**	0.77	0.28	0.63	0.53
P_3h__tend, hPa	r	−0.05	−0.00	0.02	0.04	−0.01	0.04	−0.01	−0.04
	P	0.10	0.90	0.51	0.23	0.74	0.19	0.84	0.22
Td_max, °C	r	−0.01	0.03	−0.01	**−0.10**	−0.04	−0.02	0.01	−0.02
	P	0.79	0.38	0.81	**0.002**	0.21	0.45	0.85	0.57
Td_min, °C	r	−0.02	0.03	−0.04	**−0.14**	−0.02	−0.02	0.02	−0.06
	P	0.63	0.36	0.25	**<0.001**	0.44	0.52	0.60	0.07
Td_range, °C	r	0.02	−0.01	**0.07**	**0.12**	−0.03	−0.00	−0.02	**0.09**
	P	0.67	0.82	**0.03**	**<0.001**	0.30	0.96	0.44	**0.003**
RH_max, %	r	−0.03	0.05	**0.11**	0.02	0.04	0.02	−0.01	0.05
	P	0.35	0.11	**<0.001**	0.57	0.16	0.44	0.82	0.15
RH_min, %	r	0.00	**0.09**	−0.02	−0.01	0.06	0.03	0.02	0.00
	P	0.99	**0.005**	0.45	0.87	0.08	0.30	0.59	0.99
RH_range, %	r	−0.01	**−0.07**	0.06	0.02	−0.05	−0.03	−0.02	0.02
	P	0.67	**0.02**	0.05	0.56	0.15	0.39	0.55	0.56
RR_6h_, mm	r	−0.03	0.01	−0.01	−0.03	0.02	0.03	0.05	−0.05
	P	0.29	0.64	0.82	0.43	0.62	0.42	0.13	0.14
WS_max, m/s	r	0.02	0.00	**−0.07**	0.02	−0.04	0.06	**0.07**	−0.01
	P	0.54	0.97	**0.03**	0.62	0.26	0.05	**0.04**	0.83

*r, correlation coefficient; T, daily temperature; P, air pressure; P_3h_tend, daily 3-hour pressure tendency; RH, relative air humidity; Td, dew point temperature; RR_6h_, cumulative amount of precipitation within 6 h; WS, wind speed; max, maximum daily value; min, minimum daily value; range, daily range; The significant associations are highlighted with bold*.

In both the stations, 16 (13.3%) of the weather parameters derived from the day of ACS incident were significantly correlated with ACS number. In the case of 5 (4.2%) parameters recoded in station A (T_min, T_max, Td_min, Td_range, and RH_max), correlation coefficients were higher than |0.10| (Table 3).

In both the stations, in total, 66 (18.3%) of the weather parameters recorded at least once, 1 to 3 days prior to ACS incident, were significantly correlated with the number of ACS in the given index day (Supplementary Table 3). In the case of 14 (3.9%) measurements, the correlation coefficients were higher than |0.10|, including 13 in station A in winter (T_max_1−3_, Td_max_1−3_, T_min_1−3_, Td_min_1−3_, and Td_range_3_) and one in station B in spring (WS_max_3_), while in station A in winter, the lower T_max, Td_max, T_min, Td_min, or the higher T_range and Td_range in the 3 days preceding the higher number of ACS in the index day.

In both the stations, in total, 46 (12.8%) of the weather parameters recorded at least once, 4 to 6 days prior to ACS incident, were significantly correlated with the number of ACS in the given index day. In the case of 15 (4.2%) measurements, the correlation coefficient was higher than |0.10|. All of them were found in station A, 13 in winter (T_max_4−6_, T_min_4−6_, Td_min_4−6_, Td_range_4−6_, and P_range_5_) and 2 in autumn (RH_max_5, 6_).

### Acute Coronary Syndrome Prevalence vs. Frontal Analysis

In the years 2008–2018, 58% of ACS occurred during days without any fronts as they constitute almost 60% of all the analyzed days. The remaining ACS happened on days with cold (11%) or warm (10%) fronts or during days with quasi-stationary frontal systems (9%), occluded fronts (6%), or with different fronts in the course of the self-same same day (6%).

In the whole period studied, especially in spring months, the number of ACS was the highest during days with cold or warm fronts, but a significant difference in the number of ACS was found only between warm front days and days without any front (Table 4). In summer, autumn, and winter, there were no significant differences in the median number of ACS across the different frontal scenarios. The occurrence and type of front was associated with different ACS distribution in May, August, and December (Table 4). The number of ACS in days with an occluded front was the lowest in May and August, while in December this was the highest. In the remaining months, there were no significant differences in ACS distribution.

**Table 4:** Median values of ACS in relation to the occurrence of a specific atmospheric front.

	**No front** ***N*** **= 2,339**	**Cold front** ***N*** **= 361**	**Warm front** ***N*** **= 517**	**Quasi–stationary** **front** ***N*** **= 221**	**Occluded front** ***N*** **= 341**	**Different fronts** ***N*** **= 239**	>***P*****–value^#^**
The whole period	38 (27–48)	40 (29–52)	40 (29–50)*	37 (28–49)	37 (27–48)	38 (27–51)	**0.012**
January	37 (25–48)	40 (31–51)	37 (25–48)	42 (30–49)	39 (28–52)	48 (36–62)	0.155
February	40 (29–51)	41 (29–56)	40 (27–58)	41 (32–48)	40 (31–49)	47 (33–59)	0.861
March	40 (29–48)	44 (29–55)	46 (29–56)	37 (21–57)	39 (31–46)	40 (28–52)	0.708
April	36 (25–50)	42 (37–52)	39 (30–52)	39 (36–54)	37 (30–46)	38 (28–55)	0.481
May	37 (28–48)	45 (32–50)	46 (30–54)*	34 (30–42)	30 (23–45)*	36 (30–45)	**0.015**
June	37 (26–47)	40 (33–59)	39 (28–47)	32 (27–42)	38 (28–45)	35 (25–57)	0.276
July	35 (26–46)	34 (32–44)	38 (30–46)	34 (27–46)	32 (23–41)	34 (24–51)	0.423
August	36 (27–45)	32 (27–43)	38 (27–47)	48 (26–56)	28 (20–38)*	26 (21–48)	**0.017**
September	38 (28–48)	39 (31–52)	42 (31–50)	39 (33–49)	36 (22–53)	40 (26–47)	0.805
October	40 (28–50)	41 (32–54)	46 (32–56)	39 (32–51)	45 (35–53)	37 (31–52)	0.388
November	38 (28–52)	39 (26–55)	38 (29–52)	31 (23–50)	46 (40–62)	44 (33–50)	0.284
December	34 (25–46)	36 (23–45)	37 (25–50)	28 (23–47)*	56 (44–60)*	36 (25–50)	**0.019**

*Data are shown as median (interquartile range)*,

# *ANOVA Kruskal-Wallis for differences in six groups*,

* *P < 0.05 vs. no-front days for post-hoc comparisons. The significant differences are highlighted with bold*.

### Machine Learning-Based Prediction for Acute Coronary Syndrome Prevalence

**Figure 1:**
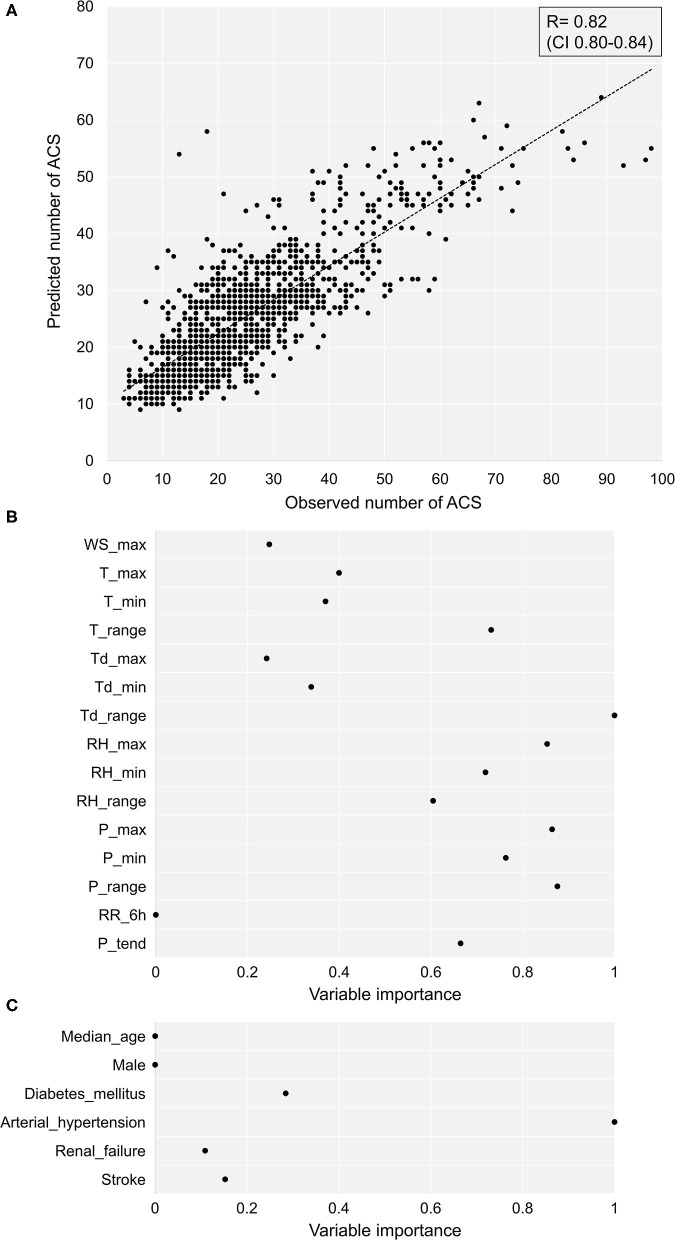
The predicted vs. observed numbers of ACS. **(A)** The correlation between the predicted vs. observed number of ACS per day. **(B)** The importance of individual weather conditions for machine learning. **(C)** The importance of clinical parameters for machine learning. ACS, acute coronary syndrome; T, temperature; P, air pressure; P_3h__tend, daily 3 hours pressure tendency, RH, relative air humidity, Td, dew point temperature, RR_6h_, precipitation, WS, wind speed, max, maximum daily value, min, minimum daily value, range, daily range.

The correlation between the predicted and observed number of ACS per day was 0.82 with a CI of 0.80–0.84 (*P* < 0.0001) (Figure 1A). Of all the weather parameters, the highest variable importance for machine learning (range 0–1) involved dew point temperature daily range, air pressure daily range and its maximum, and RH maximum with 1.00, 0.875, 0.864, and 0.853, respectively (Figure 1B). Among the clinical parameters, the variable importance for hypertension was 1.00, the variable importance for diabetes mellitus was 0.28, the variable importance for a history of stroke was 0.10, the variable importance for chronic kidney disease was 0.07, the variable importance for median age was 0, and the variable importance for sex was 0 (Figure 1C).

**Figure 2:**
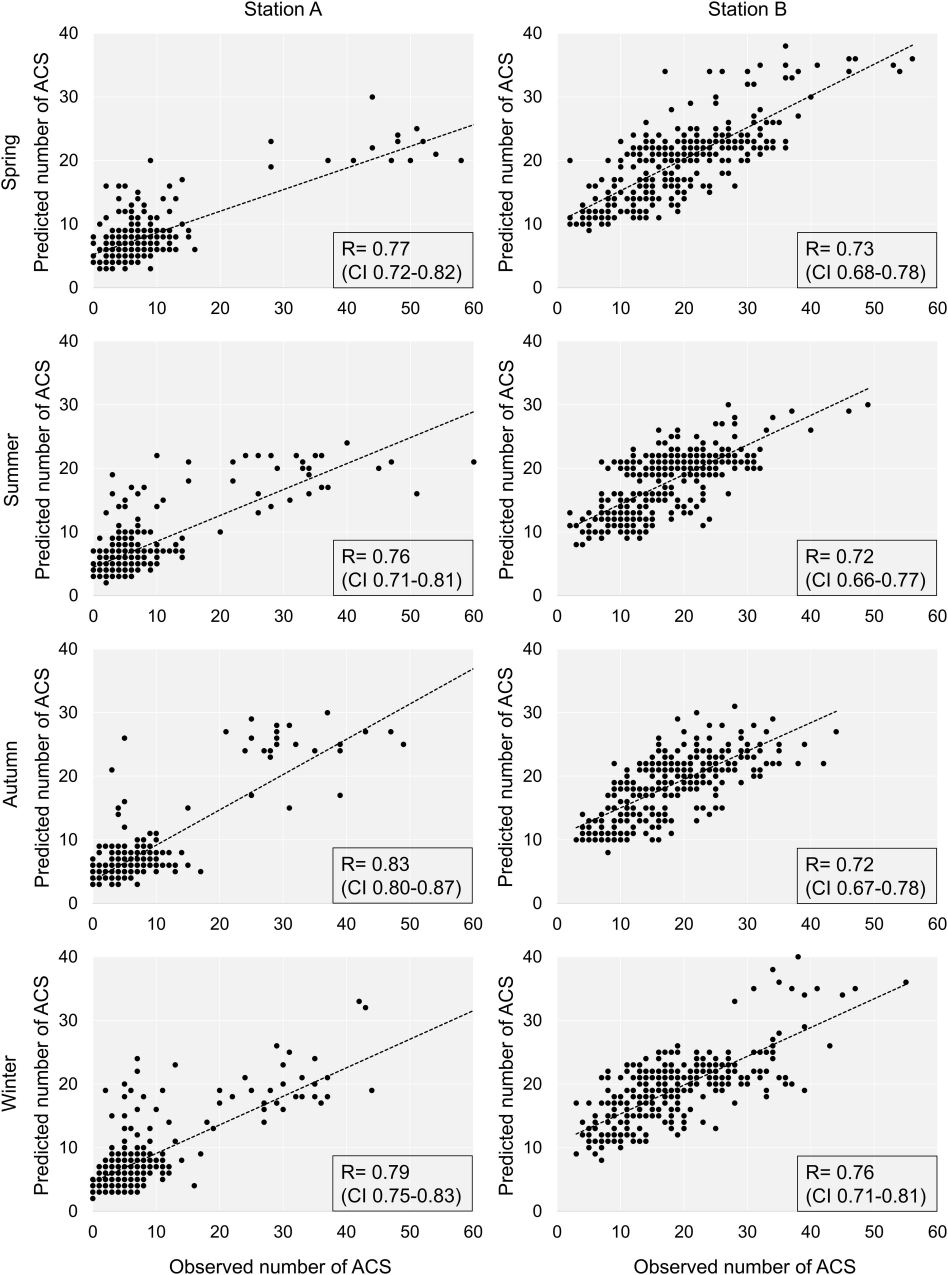
The predicted vs. observed numbers of ACS in individual seasons and meteorological stations. ACS, acute coronary syndrome.

For individual seasons, the correlations between the observed and predicted number of ACS (Figure 2) within the operations of station A were for spring 0.77 (CI 0.72–0.82), for summer 0.76 (CI 0.71–0.81), for autumn 0.83 (CI 0.80–0.87), and for winter 0.79 (CI 0.75–0.83) (*P* < 0.0001 for each correlation). The analogous correlations for station B were for spring 0.73 (CI 0.68–0.78), for summer 0.72 (CI 0.66–0.77), for autumn 0.72 (CI 0.67–0.78), and for winter 0.76 (CI 0.71–0.81) (*P* < 0.0001 for each prediction).

**Figure 3:**
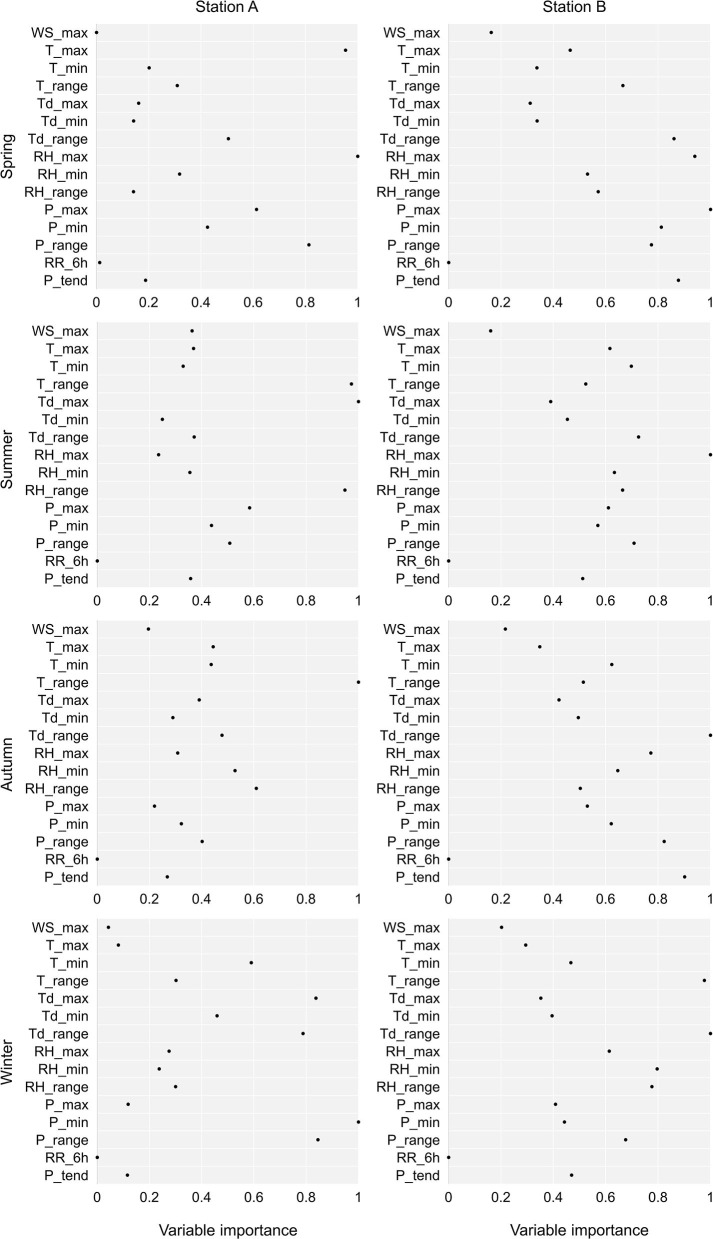
The importance of weather conditions in prediction of the number of ACS with machine learning in relation to the season and the meteorological station. T, temperature; P, air pressure; P_3h__tend, daily 3 h pressure tendency; RH, relative air humidity; Td, dew point temperature; RR_6h_, precipitation; WS, wind speed; max, maximum daily value; min, minimum daily value; range, daily range.

Of all the analyzed meteorological parameters, the most important in the machine learning model with a variable importance of more than 0.8 was for station A in spring RH_max, T_max, and P_range, in summer Td_max, T_range, and RH_range, in autumn T_range, and in winter P_min, P_range, and Td_max (Figure 3). For station B, the meteorological parameters with a variable importance of more than 0.8 were in spring P_max, RH_max, Td_range, P_3h_tend, and P_min, in summer RH_max, in autumn Td_range, P_range, and P_3h__tend, and in winter Td_range and T_range.

## Discussion

### Summary of Findings

To the best of our knowledge, this study is the first study to demonstrate that weather parameters analyzed with an artificial intelligence system using machine learning accurately predict the daily number of ACS in a temperate climate zone. The accuracy of this prediction turned out to be irrespective of the season and location of the meteorological station. Machine learning system has produced a unique prediction pattern based on a broad set of weather data including their absolute values, several hour tendencies, and also their dynamic changes derived from the day of ACS and 6 days prior to ACS. Of all the analyzed meteorological parameters in this specific population, the most influential on these predictions was both the daily ranges of dew point temperature and air pressure, as well as the maximum value of air pressure and of relative humidity. On the other hand, analysis of atmospheric fronts as well as individual weather parameters provided several univariate and significant relationships; however, ones not universal and only specific to a selected season and station. Thus, this study provides evidence indicating that artificial intelligence algorithms used for the analysis of weather conditions together might be a valuable and clinically important tool estimating the everyday risk of ACS.

### Univariate Relationships Between Weather and Acute Coronary Syndrome Prevalence

In this study, we observed significant but weak correlations between individual weather parameters and ACS prevalence. Unfortunately, these relationships were not repeatable for all the seasons and meteorological stations, thus their predictive value was useless. The WHO Monitoring Trends and Determinants in Cardiovascular Disease (MONICA) project (21) showed that a 10°C decrease was associated with a 13% increase of the total number of myocardial infarctions or coronary deaths. For an atmospheric pressure, a V-shaped relationship was found with a minimum of the daily event rate at 1,016 hPa. Simultaneously, a 10-hPa decrease below or a 10-hPa increase above 1,016 hPa was associated with a 12 or 11% increase in ACS event rate, respectively. Swedish analysis (3) indicated a higher ACS prevalence in the days with air temperatures of <0°C, while ACS rate was lower when temperatures rose to 3–4°C. Equally Canadian data (22) revealed that cold days with a temperature below the 1st percentile of temperature distribution were associated with a 29% increase in acute MI rate (95% CI 15–45) and high temperatures above the 99th percentile increased coronary heart disease hospitalizations by 6% (95% CI 1–11) as compared with the days with an optimal temperature. Low air temperatures were a significant risk factor for hospital admissions from diseases of the circulatory system also in the Iberian Peninsula, regardless of calculated meteorological index (23). Urban and Kyselý (24) have found that apparent temperature and physiologically equivalent temperature appear to be more universal predictors of heat- and cold-related mortality than UTCI when both the urban and rural environments were of concern. In turn, significant cold-related mortality in the rural region showed potential for UTCI to become a useful tool in cold exposure assessments. Michelozzi et al. (25) have analyzed the impact of high environmental temperatures on hospital admissions in 12 European cities participating in the Assessment and Prevention of Acute Health Effects of Weather Conditions in Europe project. They found that high temperatures have a specific impact on respiratory admissions, particularly in the elderly population, but the underlying mechanisms were unclear. In turn, high temperature was associated with an increased cardiovascular mortality, but not cardiovascular admissions. These relationships also remain not elucidated. In this study, the strongest univariate correlations concerned with temperature and dew point temperature only in winter in station A. These results indicate that a universal ACS prediction based on single individual weather parameters does not work. It may be due to the fact of significant day-to-day variability of weather conditions in Southern Poland, described also by Piotrowicz (26).

Given the complex nature of the weather, in the next step, we focused on the influence of atmospheric fronts on the occurrence of ACS. The median daily number of ACS was higher on days with warm fronts compared to days without any front, especially in spring. Recently, Boussoussou et al. (27) have observed in patients with acute cardiovascular diseases in the area of Budapest a 9.5% increase in hospitalizations associated with cold or occluded fronts. This effect was 10% stronger in a situation of day-to-day front replacement from warm or stationary to a cold one. In Europe, active frontal weather occurs in 40% of days in a year and frontal systems induce substantial and dynamic modifications of weather conditions, including significant air pressure and wind speed shifts, as well as temperature changes (28). Therefore, the approach to assess the number of ACS depending on the type of front seemed to be particularly justified, but again it turned out that the results were not repeatable for individual seasons as well as for shorter time intervals such as months.

### Weather Parameters Analyzed With Artificial Intelligence for Prediction of Acute Coronary Syndrome Prevalence

The lack of a satisfactory predictive value of individual weather parameters as well as frontal analysis has become an argument for applying a more sophisticated analytical method. In this study, artificial intelligence with machine learning using the Random Forest system was used to predict the number of ACS based on the weather characteristics. Ambale-Venkatesh et al. (29) have tested random survival forest to predict six cardiovascular outcomes in comparison to standard risk scores. They found that artificial intelligence added to traditional methods improves the prediction accuracy of cardiovascular events in an initially asymptomatic population. In turn, Wang et al. (30) have shown that the Random Forest displayed a satisfying performance compared to traditional linear regression models for heatstroke prediction based on meteorological and socioeconomic factors. The feature identification process of building a machine learning model that combined both the data-driven methodology and domain knowledge resulted in comprehensive variables in complex sets. Our machine learning-based analysis showed seasonal and regional differences in the influence of atmospheric processes on ACS prevalence indicating the most influential weather parameters specific to a given time and place. Weather elements act as stimuli, especially when they reach extreme values or their parameters change in a short time interval. Some of them act permanently, being intensified by synergism with others finally forming a stimulant weather complex. Thermal and moisture conditions are one of the most important factors, which profile the human heat budget. In this study, the most influential on the prevalence of ACS was maximum relative humidity and the daily range of dew point temperature describing temperatures at which the concentration of water vapor in the air was saturated. A low temperature activates the thermoreceptors of the hypothalamic-pituitary-adrenal axis and stimulates adrenal glands to secrete adrenalin, which causes vasoconstriction and blood pressure elevation. Finally, increased shear stress may induce atherosclerotic plaque rupture. Simultaneously, cold weather increases diuresis and diminishes serum volume deteriorating its rheological properties (31). In contrast, a high temperature leads to parasympathetic nervous system stimulation, vasodilatation, and a decrease in blood pressure (32). Mechanical stimuli caused, in turn, by atmospheric circulation, particularly pressure and frontal systems, fluctuations that affect the human body through air pressure and wind speed. In this study, the maximum and daily range of atmospheric pressure was found to be the most influential for a learning machine process with specific differences for separate seasons.

First, weather conditions vary across different climate zones; therefore, analysis with a machine learning model for our specific region cannot be one-to-one extrapolated to other regions, with local validation being required each time. Second, particular caution must be exercised when interpreting the results of meteorological variables with local effects, such as precipitation, even if they were included in regional analyses. Third, for patients with NSTEMI, the date of hospital admission does not precisely reflect the time of symptom onset due to the delay effect associated with the diagnosis process, while the timing of symptoms for patients with STEMI can be considered fairly accurate. Fourth, in this study, solar radiation characteristics were not analyzed. Although they are important for human body, their effects are gradual and their availability is limited what would make a study difficult for further implementation. Finally, the clinical characteristics of the studied groups are limited; hence, the National Health System has to collect data selectively.

## Conclusion

Our findings indicate that weather conditions are useful for the prediction of the number of ACS in a temperate climate zone, if analyzed with an artificial intelligence system. Simultaneously, the analysis of individual weather parameters or frontal scenarios, although separately providing weak univariate relationships, was insufficient for a reproducible prognosis in different seasons and locations. The adoption of these novel findings in other climatic zones might be clinically relevant, but would require local validation.

## Data Availability Statement

The original contributions presented in the study are included in the article/Supplementary Material, further inquiries can be directed to the corresponding author/s.

## Ethics Statement

The studies involving human participants were reviewed and approved by Bioethics Committee of the Jagiellonian University. Written informed consent for participation was not required for this study in accordance with the national legislation and the institutional requirements.

## Author Contributions

AW, PM, and JZ: conception. AW and PM: data collection and manuscript drafting. BB and JZ: data analysis. BB, AW, JN, and JZ: data interpretation and revision of the manuscript. All authors contributed to the article and approved the submitted version.

## Conflict of Interest

The authors declare that the research was conducted in the absence of any commercial or financial relationships that could be construed as a potential conflict of interest.

## Publisher's Note

All claims expressed in this article are solely those of the authors and do not necessarily represent those of their affiliated organizations, or those of the publisher, the editors and the reviewers. Any product that may be evaluated in this article, or claim that may be made by its manufacturer, is not guaranteed or endorsed by the publisher.
