# Supplementary material for: Machine Learning Analyzed Weather Conditions as an Effective Means in the Predicting of Acute Coronary Syndrome Prevalence
Source: Front Cardiovasc Med. 2022 Apr 8;9:830823. doi: 10.3389/fcvm.2022.830823 (PMC9024050; doi:10.3389/fcvm.2022.830823)

Supplementary Material

# Supplementary Table 1. The distribution of patients in the studied period.

| Year | All ACS patients | UA | STEMI | NSTEMI |
| --- | --- | --- | --- | --- |
| 2008 | 11826 | 6993(59.1) | 2757(23.3) | 2076(17.6) |
| 2009 | 10316 | 5821(56.4) | 2527(24.5) | 1968(19.1) |
| 2010 | 8910 | 4564(51.2) | 2189(24.6) | 2157(24.2) |
| 2011 | 11657 | 7076(61.0) | 1848(15.9) | 2733(23.4) |
| 2012 | 14500 | 9861(68.0) | 1660(11.4) | 2979(20.5) |
| 2013 | 8877 | 4213(47.5) | 1637(18.4) | 3027(34.1) |
| 2014 | 8849 | 4124(46.6) | 1497(16.9) | 3228 (36.5) |
| 2015 | 8487 | 3793(44.7) | 1589(18.7) | 3105(36.6) |
| 2016 | 8030 | 3770(46.9) | 1367(17.0) | 2893(36.0) |
| 2017 | 7819 | 3304(42.3) | 1503(19.2) | 3012(38.5) |
| 2018 | 6631 | 2267(34.2) | 1468(22.1) | 2896(43.7) |

# Abbreviations: data are shown as numbers (percentages), UA: unstable angina, STEMI: ST elevation myocardial infarction, NSTEMI: non-ST elevation myocardial infarction.

# Supplementary Table 2. The distribution of patients according to the day of the week.

|  | Monday  N=20768 | Tuesday  N=19084 | Wednesday  N=18114 | Thursday  N=16825 | Friday  N=13767 | Saturday  N=7508 | Sunday  N=9836 | P-value |
| --- | --- | --- | --- | --- | --- | --- | --- | --- |
| Age | 67(59-76) | 68(60-76) | 68(60-76) | 68(60-77) | 68(59-77) | 69(60-79) | 69(60-77) | <0.001 |
| Male | 13195(63.5) | 11863(62.2) | 11157(61.6) | 10287(61.1) | 8391(61.0) | 4631(61.7) | 6335(64.4) | <0.001 |
| UA | 12171(58.6) | 11135(58.3) | 10562(58.3) | 9124(54.3) | 6532(47.4) | 2099(28.0) | 4163(42.3) | <0.001 |
| STEMI | 3465(16.7) | 3027(15.9) | 2884(15.9) | 3218(19.1) | 2809(20.4) | 2306(30.7) | 2333(23.7) | <0.001 |
| NSTEMI | 5132(24.7) | 4922(25.8) | 4668(25.8) | 4483(26.6) | 4426(32.1) | 3103(41.3) | 3340(34.0) | <0.001 |
| Comorbidities | | | | | | | | |
| Diabetes mellitus | 7215(34.7) | 6747(35.4) | 6358(35.1) | 5838(34.7) | 4970(36.1) | 2761(36.8) | 3530(35.9) | 0.006 |
| Arterial hypertension | 14791(71.2) | 13471(70.6) | 12817(70.8) | 12055(71.6) | 10133(73.6) | 5449(72.6) | 6702(68.1) | <0.001 |
| Renal failure | 944(4.5) | 842(4.4) | 812(4.5) | 706(4.2) | 605(4.4) | 405(5.4) | 484(4.9) | 0.002 |
| Stroke | 1425(6.9) | 1286(6.7) | 1211(6.7) | 1119(6.7) | 906(6.6) | 604(8.0) | 660(6.7) | 0.002 |
| Outcome | | | | | | | | |
| In-hospital death | 758(3.6) | 709(3.7) | 703(3.9) | 678(4.0) | 638(4.6) | 535(7.1) | 628(6.4) | <0.001 |

Abbreviations: data are shown as number (%), UA: unstable angina, STEMI: ST elevation myocardial infarction, NSTEMI: non-ST elevation myocardial infarction. P-value for differences between days of the week.

# Supplementary Table 3. The highest recorded correlations between the number of ACS and the weather conditions as measured one to three days prior to the day of ACS.

|  | | Station A | | | | Station B | | | |
| --- | --- | --- | --- | --- | --- | --- | --- | --- | --- |
|  |  | Spring | Summer | Autumn | Winter | Spring | Summer | Autumn | Winter |
| T_max °C | r | -0.03 | -0.05 | 0.02 | **-0.13** | -0.04 | **-0.09** | -0.04 | 0.02 |
|  | P | 0.36 | 0.07 | 0.58 | **<0.001** | 0.26 | **0.004** | 0.20 | 0.50 |
| T_min_,_ °C | r | -0.02 | 0.00 | **-0.09** | **-0.17** | -0.05 | **-0.08** | **-0.08** | -0.05 |
|  | P | 0.50 | 0.73 | **0.03** | **<0.001** | 0.09 | **0.01** | **0.006** | 0.14 |
| T_range_,_ °C | r | -0.03 | **-0.07** | **0.09** | **0.10** | 0.04 | -0.05 | 0.05 | **0.08** |
|  | P | 0.33 | **0.03** | **0.006** | **0.003** | 0.17 | 0.06 | 0.12 | **0.004** |
| P_max, hPa | r | 0.03 | -0.05 | 0.05 | **0.07** | -0.03 | -0.06 | -0.05 | 0.03 |
|  | P | 0.30 | 0.09 | 0.09 | **0.03** | 0.38 | 0.43 | 0.14 | 0.35 |
| P_min, hPa | r | 0.02 | -0.06 | 0.06 | **0.07** | -0.01 | **-0.08** | 0.04 | 0.04 |
|  | P | 0.54 | 0.06 | 0.08 | **0.04** | 0.69 | **0.008** | 0.17 | 0.22 |
| P_range, hPa | r | 0.02 | **0.06** | -0.02 | -0.03 | -0.04 | 0.06 | -0.06 | -0.03 |
|  | P | 0.51 | **0.04** | 0.52 | 0.30 | 0.25 | 0.08 | 0.08 | 0.28 |
| P_3h__tend, hPa | r | 0.02 | 0.04 | 0.02 | 0.04 | 0.01 | 0.04 | **-0.09** | -0.03 |
|  | P | 0.44 | 0.21 | 0.58 | 0.27 | 0.71 | 0.19 | **0.003** | 0.34 |
| Td_max, °C | r | -0.03 | 0.03 | -0.06 | **-0.14** | -0.04 | -0.06 | -0.04 | -0.01 |
|  | P | 0.36 | 0.36 | 0.08 | **<0.001** | 0.22 | 0.08 | 0.22 | 0.76 |
| Td_min, °C | r | -0.05 | 0.04 | **-0.07** | **-0.17** | -0.05 | -0.04 | -0.06 | -0.04 |
|  | P | 0.15 | 0.22 | **0.03** | **<0.001** | 0.09 | 0.18 | 0.07 | 0.26 |
| Td_range, °C | r | 0.04 | -0.03 | 0.03 | **0.16** | 0.04 | 0.05 | 0.04 | 0.07 |
|  | P | 0.16 | 0.31 | 0.31 | **<0.001** | 0.19 | 0.11 | 0.18 | 0.02 |
| RH_max, % | r | 0.06 | 0.06 | **0.07** | 0.02 | 0.08 | 0.06 | **0.07** | 0.02 |
|  | P | 0.09 | 0.05 | **0.04** | 0.53 | 0.007 | 0.05 | **0.03** | 0.61 |
| RH_min, % | r | 0.02 | **0.08** | -0.03 | -0.04 | -0.01 | **0.07** | -0.08 | -0.06 |
|  | P | 0.45 | **0.01** | 0.23 | 0.27 | 0.79 | **0.02** | 0.22 | 0.06 |
| RH_range, % | r | -0.01 | **-0.06** | 0.06 | 0.04 | 0.03 | -0.06 | -0.03 | 0.06 |
|  | P | 0.78 | **0.03** | 0.05 | 0.27 | 0.34 | 0.05 | 0.37 | 0.05 |
| RR_6h_, mm | r | -0.05 | 0.03 | **-0.09** | **-0.07** | -0.05 | **0.07** | **-0.09** | **-0.09** |
|  | P | 0.11 | 0.26 | **0.004** | **0.03** | 0.12 | **0.02** | **0.003** | **0.003** |
| WS_max, m/s | r | 0.03 | -0.03 | -0.06 | -0.04 | **-0.12** | **0.07** | **-0.08** | **-0.07** |
|  | P | 0.31 | 0.39 | 0.06 | 0.17 | **<0.001** | **0.04** | **0.003** | **0.03** |

Abbreviations: r: correlation coefficient, T: daily temperature, P: air pressure, P_3h_tend: daily 3-hour pressure tendency, RH: relative air humidity, Td: dew point temperature, RR_6h_: cumulative amount of precipitation within 6 hours, WS: wind speed, max: maximum daily value, min: minimum daily value, range: daily range. The significant associations are highlighted with bold.

# Supplementary Figure 1. The region of interest.


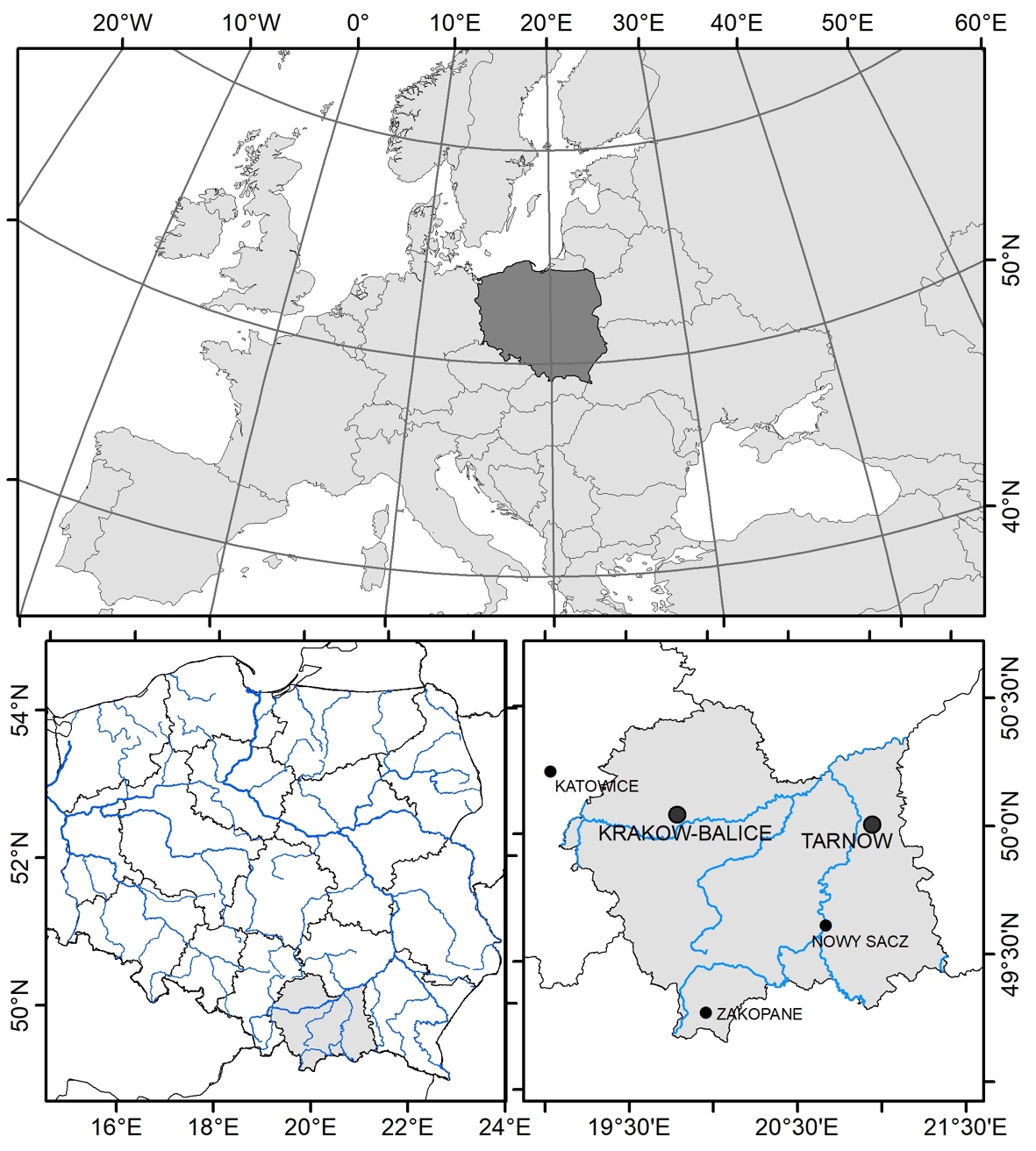


# Supplementary Figure 2. The distribution of patients in the annual cycle in the years 2008-2018.


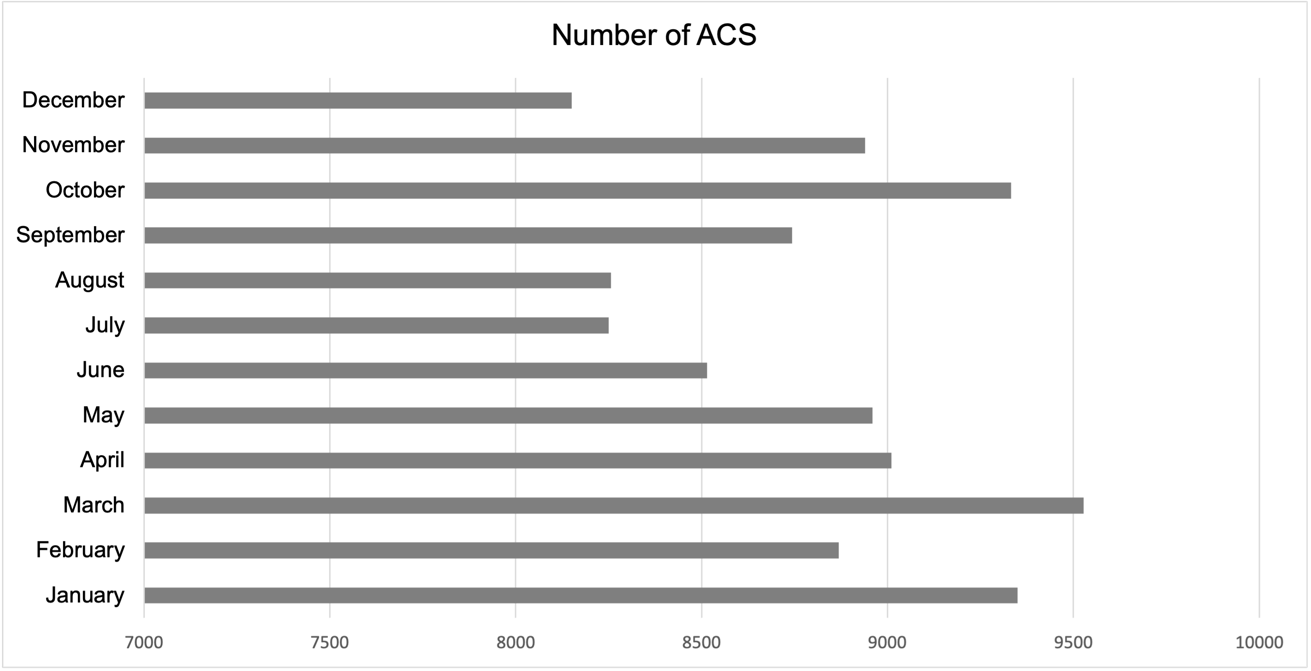

Supplement: Supplementary file 1 [file Data_Sheet_1.docx]
